# P-556. Efficacy and Safety of Bictegravir/Emtricitabine/TenofovirAlafenamide in Females Living With HIV: 96 Weeks Real Life Analysis

**DOI:** 10.1093/ofid/ofae631.755

**Published:** 2025-01-29

**Authors:** Andrea De Vito, Manuela Ceccarelli, Agnese Colpani, Emmanuele Venanzi Rullo, Serena Spampinato, Sonia Agata Sofia, Grazia Pantò, Antonio Albanese, maria Chiara Frasca, Michele Salvatore Paternò Raddusa, Antonio Edoardo Campanella, Laura Santoro, Ylenia Russotto, laura todaro, Mariagiovanna Coco, Cristina Micali, Sarah Pulvirenti, claudia calì, Maria Antonietta Di Rosolini, Giulia moi, giuseppe conti, Andrea Marino, Giovanni Francesco Pellicanò, Carmelo Iacobello, Arturo Montineri, Bruno Santi Cacopardo, goffredo angioni, Giordano Madeddu, Giuseppe Nunnari

**Affiliations:** University of Sassari, Sassari, Sardegna, Italy; University of Enna KORE, Enna, Sicilia, Italy; University of Sassari, Sassari, Sardegna, Italy; Unit of Infectious Diseases, Department of Clinical and Experimental Medicine, University of Messina, 98124 Messina, Italy, Messina, Sicilia, Italy; University of Messina, Catania, Sicilia, Italy; Azienda Ospedaliera per l'Emergenza Cannizzaro, Catania, Sicilia, Italy; Azienda Ospedaliera per l'Emergenza Cannizzaro, Catania, Sicilia, Italy; "Papardo" Hospital, Unit of Infectious Diseases, Messina, Italy,, messina, Sicilia, Italy; G. Rodolico - S. Marco" University Hospital, Unit of Infectious Diseases, Catania, Italy, Catania, Sicilia, Italy; University of Messina, Catania, Sicilia, Italy; University of Messina, Catania, Sicilia, Italy; University of Messina, Catania, Sicilia, Italy; Unit of Infectious Diseases, Department of Clinical and Experimental Medicine, University of Messina, 98124 Messina, Italy, Messina, Sicilia, Italy; University of Messina, Catania, Sicilia, Italy; University of Messina, Catania, Sicilia, Italy; University of Messina, Catania, Sicilia, Italy; University of Messina, Catania, Sicilia, Italy; University of Messina, Catania, Sicilia, Italy; Unit of Infectious Diseases, Giovanni Paolo II Hospital, Ragusa, ragusa, Sicilia, Italy; UNIVERSITY OF SASSARI, SASSARI, Sardegna, Italy; University of Messina, Catania, Sicilia, Italy; University of Catania, Messina, Sicilia, Italy; Unit of Infectious Diseases, Department of Adult and Childhood Human Pathology “Gaetano Barresi”, University of Messina, 98124 Messina, Italy, Messina, Sicilia, Italy; University of Catania, Messina, Sicilia, Italy; G. Rodolico - San Marco Hospital, Catania, Sicilia, Italy; University of Catania, Messina, Sicilia, Italy; Unit of Infectious Diseases, Ospedale Santissima Trinità, ASL8 Cagliari, Italy, Cagliari, Sardegna, Italy; University of Sassari, Sassari, Sardegna, Italy; University of Catania, ARNAS Garibaldi" Hospital, Unit of Infectious Diseases, Catania, Italy, Catania, Sicilia, Italy

## Abstract

**Background:**

Females with HIV (FWH) are underrepresented in HIV trials. Pooled data from 5 trials at 48 weeks demonstrated the virological efficacy and safety of bictegravir/emtricitabine/tenofovir alafenamide (B/F/TAF). We evaluated the safety and efficacy of B/F/TAF in a cohort of 99 FWH, in a real-life setting, at 48 and 96 weeks from the switch.

**Figure:**
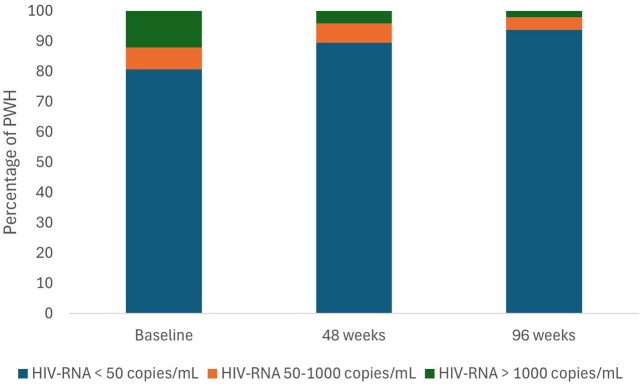

**Methods:**

We conducted a multicentric cohort study that included all experienced FWH starting B/F/TAF treatment in the Shine&Shic cohort across six Infectious Disease Centers in Italy. We collected demographic, epidemiological, clinical, and laboratory data at the time of the switch and at 48 and 96 weeks. Informed consent was obtained from all participants.

Data were reported as numbers (%), or using median and interquartile range (IQR), as appropriate. We assessed differences between baseline, 48 weeks, and 96 weeks using the Mann-Whitney test. A p-value of less than 0.05 was considered significant.

**Figure:**
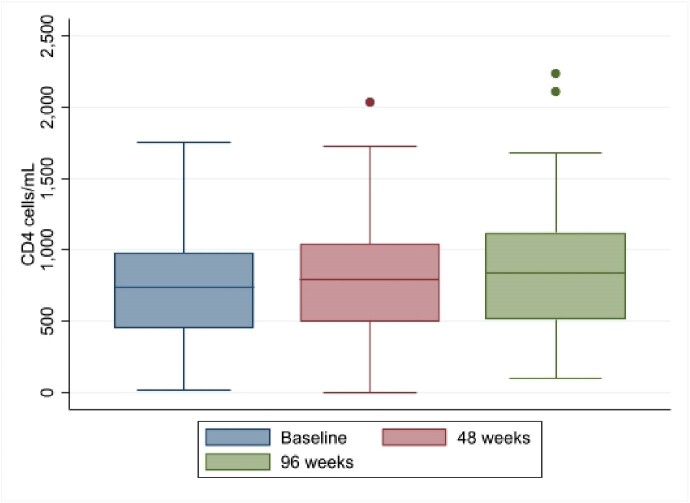

**Results:**

We included 99 females with HIV (FWH) in the study, with a median age of 51.9 years (IQR 43.1-57.8) and a duration of HIV infection of 15.1 years (IQR 5.1-24.7). During follow-up, we observed an increase in the percentage of FWH with an undetectable viral load (Figure 1).

We noted a significant increase in CD4 cells/mL and in CD4/CD8 ratio at 48 and 96 weeks and no differences in CD8 cell counts (Table 2).

Of importance, we observed a significant reductions in total cholesterol and low-density lipoprotein (LDL) at both 48 and 96 weeks. No significant changes were observed in transaminase levels, glucose levels, high-density lipoprotein (HDL), or triglycerides (Table 2). Regarding safety, there was a significant increase in creatinine at 48 and 96 weeks.

We registered 9 interruptions of B/F/TAF: 3 due to loss to follow-up, 1 death, 1 switch to long-acting treatment, 1 due to adverse events, and in 3 cases, due to patient’s choice.

**Figure:**
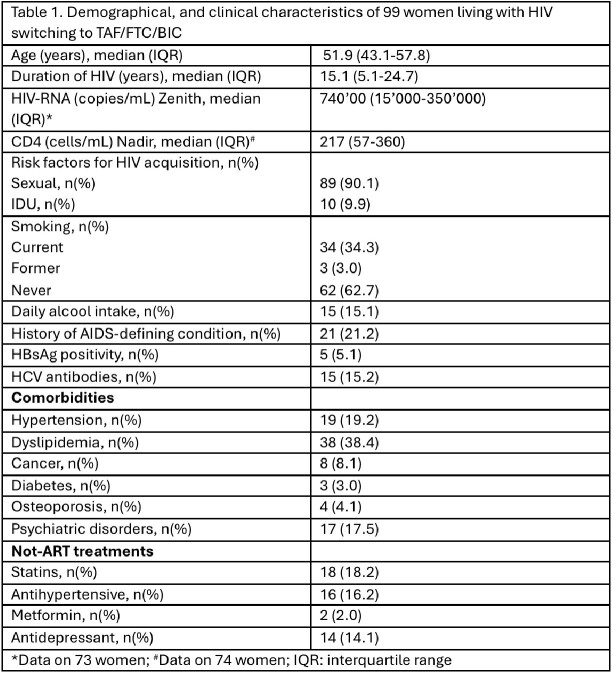

**Conclusion:**

The number of FWH on B/F/TAF under virological suppression increased at 48 and 96 weeks. Furthermore, CD4 T-cell and CD4/CD8 ratio also augmented, underlining an amelioration of the immunological and inflammatory profile. Of note, LDL cholesterol levels significantly decreased overtime. B/F/TAF is virologically and immunologically effective in FWH, in a real-life setting, at 96 weeks from the switch.

**Figure:**
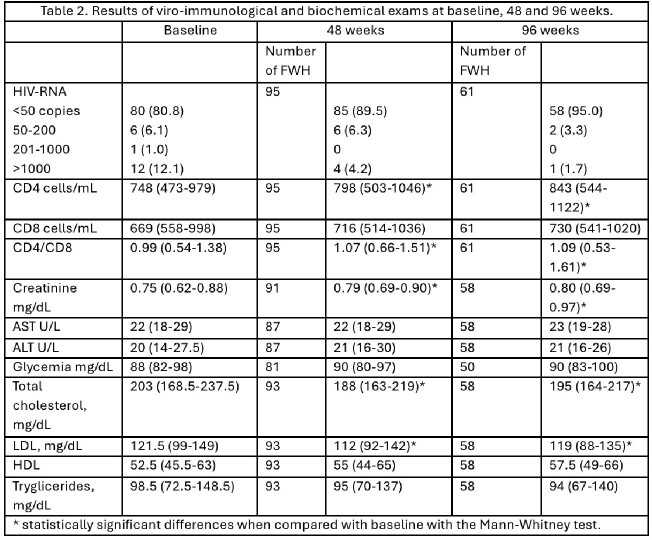

**Disclosures:**

**All Authors**: No reported disclosures

